# A randomised trial of the effectiveness of instructor versus automated manikin feedback for training junior doctors in life support skills

**DOI:** 10.1007/s40037-020-00631-y

**Published:** 2020-11-26

**Authors:** Chris Wilson, Erin Furness, Leah Proctor, Greg Sweetman, Kathryn Hird

**Affiliations:** 1 Medical Education Unit, Fiona Stanley Fremantle Hospitals Group, Fiona Stanley Hospital, Murdoch, WA Australia; 2 School of Medicine, University of Notre Dame Australia, Fremantle, WA Australia

**Keywords:** Hospital life support, Basic life support, Cardiac compressions, Ventilation, Simulation, Audio-visual feedback

## Abstract

**Introduction:**

Australian Standards require that clinicians undergo regular training in skills required to respond to the acute deterioration of a patient. Training focuses on the ability to appropriately respond to cardiac arrest, including delivering cardiac compressions, ventilation and appropriate defibrillation. Providing such training comes at a significant cost to the organisation and impacts on clinician time in direct patient care. If effective, the use of an automated manikin could significantly reduce costs and provide consistent training experiences.

**Methods:**

Fifty-six resident medical officers were randomised to two groups to test two skills components of hospital life support training under two feedback conditions. The skills components were cardiac compressions and bag-valve-mask ventilation. The feedback conditions were automated feedback delivered by a simulation manikin and traditional feedback delivered by an instructor. All participants were exposed to both skills components and both feedback conditions in a counterbalanced block design. Participants completed surveys before and after training.

**Results:**

The results demonstrated significantly better performance in cardiac compressions under the automated manikin feedback condition compared with the instructor feedback condition. This difference was not observed in bag-valve-mask ventilation. The majority of participants found the automated manikin feedback more useful than the instructor feedback.

**Discussion:**

Automated manikin feedback was not inferior to instructor feedback for skill acquisition in cardiac compressions training. The automated feedback condition did not achieve the same level of significance in bag-valve-mask ventilation training. Results suggest training with automated feedback presents a cost-effective opportunity to lessen the training burden, whilst improving skill acquisition.

**Electronic supplementary material:**

The online version of this article (10.1007/s40037-020-00631-y) contains supplementary material, which is available to authorized users.

## Introduction

During cardiac arrest, cardiac compressions, bag-valve-mask (BVM) ventilation and appropriate defibrillation are critical healthcare skills. Patient outcomes have been shown to be improved when high quality cardiopulmonary resuscitation (CPR) is combined with early defibrillation [1]. Hospital life support training covers these skills and is a yearly mandatory training requirement for all clinical staff in Western Australian hospitals as part of the Australian Commission on Safety and Quality in Health Care’s National Safety and Quality Health Service (NSQHS) Standards [2]. Despite the obvious importance of regular training in hospital life support, there is limited knowledge concerning the efficacy of teaching methods, learning and retention of skills [3]. Across Europe and North America, traditional course concepts are being challenged with the introduction of new approaches to improve efficiency and cost-effectiveness of training. The overall aim is to reduce the time spent in education for both hospital workers and responsible educators. One teaching innovation involves the integration of teaching into everyday activities allowing shorter lessons and multiple repetitions to improve retention of information and skill base [4]. In the hospital life support setting, one way to achieve this goal is to provide short individualised CPR self-learning with automated assessment and feedback. Some evidence exists that retention of cardiac compression skills is limited to 6 months after training [5]. It is envisaged that with the use of automated simulation manikins, training programs can offer on-demand training without requiring the presence of skilled human instructors. By making these automated manikins available in the work environment, the ease of access will increase training opportunities and promote skill retention, while limiting the loss of direct patient care to mandatory training sessions and the associated staffing cost.

In a large quaternary teaching hospital of approximately 1100 doctors in Perth, Western Australia, the current model of hospital life support training is instructor-intensive, with an individual instructor required to assess three doctors’ performance of the different skills simultaneously. Assessment is based on the experience of the credentialed instructor, who utilises an assessment form containing standardised assessment criteria that is consistent with contemporary practice. As the instructor assessment is subjective, and no interrater reliability measures are routinely taken, this model leaves the potential for suboptimal skills to go unchecked. In addition, there is no capacity to monitor the acquisition of skills and degradation of skills over time as no detailed records of performance are kept.

Laerdal Medical’s Resuscitation Quality Improvement^TM^ (RQI) mobile simulation station (Laerdal Medical Corporation, Stavanger, Norway) utilises an automated manikin to provide participants with one-on-one, immediate, standardised feedback correcting suboptimal technique or reinforcing good technique. This feedback is in response to performance of cardiac compressions and BVM ventilation based on various parameters, with Laerdal Medical’s published target of 75% required to pass the assessment for each skill. As a global company that develops products and programs for healthcare providers to improve patient outcomes and survival, Laerdal Medical’s target has been widely accepted in the setting of hospital life support training.

This study explored the efficacy of real-time audio-visual automated feedback delivered by the RQI compared with traditional feedback delivered by an instructor for the acquisition of hospital life support skills for junior doctors by analysing performance data recorded by the RQI under both feedback conditions. It is hypothesised that junior doctors’ performance in delivering CPR utilising automated manikin feedback will be closer to Laerdal Medical’s published target of 75% than their performance in the same skills utilising instructor feedback.

## Methods

### Participants

Fifty-six resident medical officer (RMO) participants were recruited during the induction phase of their employment in a large quaternary teaching hospital in Perth, Western Australia. The RMOs were commencing employment at the hospital as part of the annual mid-year intake of junior doctors. The period of recruitment for the study commenced when the RMOs were enrolled in the hospital orientation program, and concluded at the commencement of the hospital orientation program. Each RMO was given the opportunity to opt out of the study, but would remain in the hospital orientation program required for all new employees. No one opted out of the study. All participants undertook hospital life support training, including cardiac compressions and BVM ventilation skills, as part of the hospital orientation program. All participants had previously received training in delivery of cardiac compressions and BVM ventilation as part of their medical training and during previous hospital-based employment.

### Experimental resources

The Laerdal Medical RQI, software version 5.2.1.51, was utilised for the study. Two medical education registrars credentialed in the facilitation of advanced life support training were utilised for the instructor feedback condition of the study. The instructors used an assessment form containing standardised assessment criteria, and threshold parameters had been agreed on by both instructors prior to the study.

### Experimental protocol

**Fig. 1:**
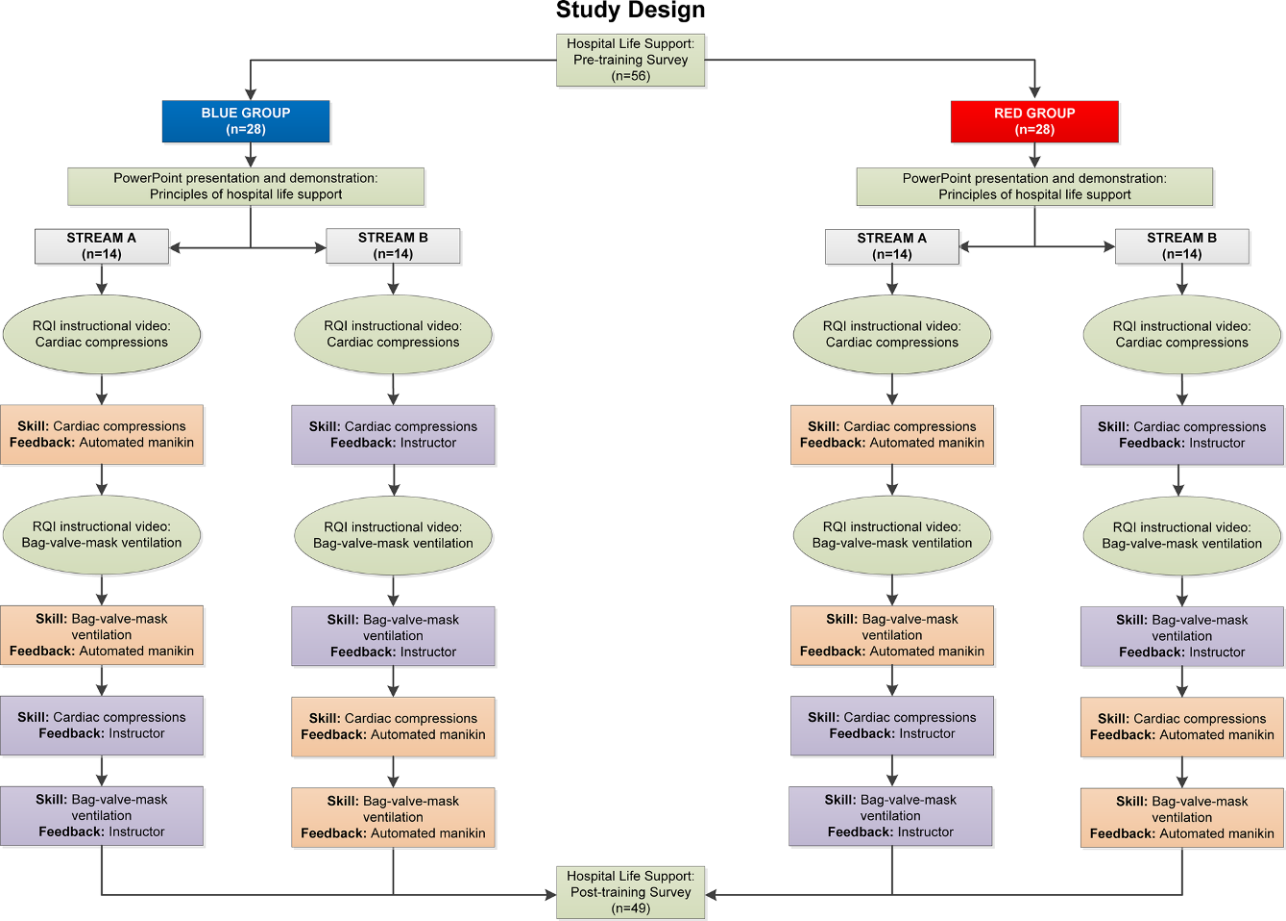
Study design

The study design involved a counterbalanced block design. See Fig. 1 for a visual representation of the study design.

Participants were randomly allocated evenly through alphabetical order of their surnames to two groups (Blue Group or Red Group) in accordance with Consolidated Standards of Reporting Trials (CONSORT) guidelines. Participants in each group were then randomly allocated using the same method to two streams (Stream A or Stream B). The randomisation, enrolment of participants and assigning of participants to interventions was actioned by the Simulation Education Officer and the Medical Education Officer involved in the coordination of hospital life support training and the hospital orientation program. All participants in the Blue Group completed their hospital life support training in the morning and all participants in the Red Group completed their hospital life support training in the afternoon. This was due to resource limitations based on the maximum number of RQIs and medical education registrars available to conduct the training at any one time.

All participants received didactic teaching on advanced life saving techniques as part of the mandatory hospital life support training provided during the hospital orientation program within their respective groups. This included instruction in and demonstration of correct technique and rate of delivery of cardiac compressions and BVM ventilation as per Australian Resuscitation Council guidelines [6]. All participants then underwent cardiac compressions and BVM ventilation skill training using a RQI under two feedback conditions. Condition 1 involved real-time audio-visual automated feedback delivered by a RQI with an observer present and took approximately 10 minutes per participant. Condition 2 involved traditional feedback delivered by an instructor and took approximately 10 minutes per participant. Stream A undertook Condition 1 first and Condition 2 second. Stream B completed the reverse presentation order. Cardiac compression skills were assessed for a period of 60 seconds under each feedback condition. BVM ventilation skills were assessed for a period of 75 seconds under each feedback condition. Each skill was assessed separately and not part of a compression-ventilation cycle.

Counterbalancing of training condition to control for the order of condition presentation was undertaken. The between-group comparison condition was automated manikin feedback versus instructor feedback. The repeated measure condition had two levels; cardiac compressions training and BVM ventilation training.

### Pre-training and post-training surveys

All participants completed a pre-training survey inquiring about the timing, frequency and nature of previous training in cardiac compressions and BVM ventilation along with current level of confidence in delivery of the skills. See Appendix A in the Electronic Supplementary Material for the pre-training survey.

Following completion of the training, participants completed a post-training survey to reassess levels of confidence and perceptions of the automated manikin feedback. See Appendix B in the Electronic Supplementary Material for the post-training survey.

### Data collection and analysis

Data from the pre- and post-training surveys were collated and analysed descriptively. Data automatically collected from the RQI included:

1. Cardiac compressions: number per cycle, hand position (%), average depth (mm), average rate (compressions per minute), compression depth (%), correct rate (%) and full recoil (%).
2. BVM ventilation: correct volume (%), average volume (%), average rate (ventilations per minute).

Statistical analysis of the performance data was achieved using independent t tests and paired t tests.

### Performance feedback

Performance under the automated manikin feedback condition was assessed according to the accuracy of cardiac compressions and BVM ventilation, which was determined by Laerdal Medical with reference to the following parameters:

1. Cardiac compressions: depth should be between 38 and 51 mm, rate should be between 100 and 120 compressions per minute, 33% or more of the duty cycle should be compressions, less than 3 kg of force should be applied on chest in the release phase.
2. BVM ventilation: patient chest rise indicates adequate ventilation volume, a surrogate measure of ventilation force was calculated by the RQI using a change in electrical resistance, inflation time should be between 0.8 and 2 seconds, ventilation rate should be between 9 and 16 ventilations per minute when more than 60 seconds since last compression.

When performance did not meet the criteria, the RQI provided relevant feedback according to these parameters, with Laerdal Medical’s published target of 75% required to pass the assessment for each skill. See Appendix C in the Electronic Supplementary Material for the documented automated manikin feedback phrases provided by the RQI.

Performance under the instructor feedback condition was assessed as per the usual hospital life support training program and the results of cardiac compressions and BVM ventilation were recorded as a pass or a fail with reference to the following assessment criteria:

1. Cardiac compressions: hands should be in the correct position in the centre of the chest and the lower half of the sternum, depth should be 5 cm or one third of the depth of the chest, rate should be between 100 and 120 compressions per minute, trainee should avoid interruptions to the compressions.
2. BVM ventilation: effective head tilt/chin lift manoeuvre and maintains good seal, aiming for 10 breaths per minute, volume between 400 and 500 ml per breath.

Both corrective and positive feedback was provided by the respective instructor, with threshold parameters which had been agreed on by both instructors prior to the study. See Appendix D in the Electronic Supplementary Material for the Hospital Life Support Assessment Form.

## Results

Fifty-six RMOs participated in this study. Thirty-one were male (55.4%). The majority of participants (61%) were aged between 25 and 29 years, with 30% under 25 years and the remainder over 30 years of age. The majority (92%) were in their second postgraduate year and the remainder were in their third postgraduate year of training. All participants had received training in advanced life support and acute care prior to being included in this study. Forty-eight percent reported previous training between 7 and 12 months prior to participation for both cardiac compressions and BVM ventilation. Thirty-six participants (64.3%) felt very confident in conducting cardiac compressions prior to the training, whereas only 20 participants (35.7%) felt very confident in carrying out BVM ventilation. Male participants were in general more confident in both compressions and ventilation than female participants, but this difference was not statistically significant. Thirty percent reported that they had used a voice advisory manikin that provided feedback in their previous training.

Independent t tests were conducted to determine if the order of training under the two feedback conditions had any impact on the participant performance scores. There were no differences for cardiac compressions under the instructor feedback condition (*t* [51] = 0.75, *p* *=* 0.45), cardiac compressions under the automated manikin feedback condition (*t* [51] = −0.1, *p* *=* 0.91), BVM ventilation under the instructor feedback condition (*t* [51] = 1.76, *p* = 0.08) and BVM ventilation under the automated manikin feedback condition (*t* [51] = 0.42, *p* *=* 0.67).

Independent t tests were conducted to determine if the instructor had any impact on the participant performance scores. There were no differences for cardiac compressions (*t* [51] = 1.28, *p* *=* 0.20) and BVM ventilation (*t* [51] = −0.74, *p* *=* 0.47).

**Fig. 2:**
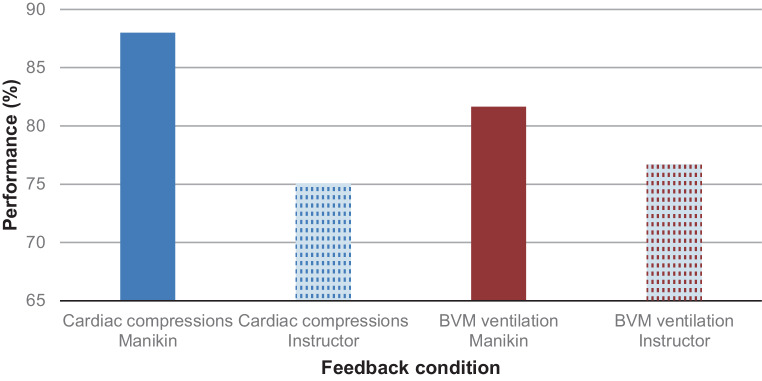
Average performance in cardiac compressions and bag-valve-mask (BVM) ventilation under automated manikin and instructor feedback conditions

Given that the order of training and instructor did not have any impact on participant performance scores, the differences between the two feedback conditions were investigated using paired t tests. The results showed that there was a consistently significantly higher rate of cardiac compressions under the automated manikin feedback condition (*x* = 88.00) compared with the instructor feedback condition (*x* = 75.81), (*t* [52] = 4.14, *p* < 0.000). The pattern of consistent performance that met or exceeded the 75% target was not observed in BVM ventilations under the automated manikin feedback condition (*x* = 81.66) compared with the instructor feedback condition (*x* = 76.7), (*t* [52] = −1.44, *p* = 0.15). It is interesting to note, however, that under both feedback conditions, the average performance scores exceeded Laerdal Medical’s published target of 75% for both cardiac compressions and BVM ventilation. See Fig. 2 for the average performance in cardiac compressions and BVM ventilation under each feedback condition.

All participants passed both skills under both feedback conditions, which negated the need for any participant to be reassessed later in the day.

Seven participants did not complete the post-training survey. Of those who did, 66% found the automated manikin feedback more useful than the instructor feedback. Only five participants found the instructor feedback more useful, and a further seven participants were ambivalent. There were no significant differences in participant confidence ratings before and after training for demonstrating cardiac compressions (*χ* [4] = 5.153, *p* *=* 0.27) or BVM ventilation (*χ* [6] = 10.46, *p* *=* 0.10).

## Discussion

Training of clinical staff in high-quality CPR is critical to patient survival from cardiac arrest [1], and forms part of the Australian NSQHS Standards [2]. Training is traditionally resource intensive, requiring skilled instructors, space and equipment in addition to time away from direct patient care for clinicians. Previous studies report that yearly skills refresher training does not match the timeline of skill degradation if those skills have been unused, as may be the case for clinicians in areas where CPR is an infrequent event [7].

The results of this study demonstrate that the psychomotor skills required for effective life support techniques such as cardiac compression depth, rate of compressions and release from cardiac compressions are effectively instructed by an automated manikin. This study showed that skill acquisition with continuous real-time audio-visual automated feedback is not inferior when compared with a human instructor for cardiac compressions training. Results support the suggestion that training of the psychomotor component of cardiac compressions could be reliably assigned to a simulation station. This result is important, as access to automated manikins offers the potential for more frequent training at a reduced cost [8].

The interconnectivity with a training database is an added benefit of the automated system. It yields additional information in monitoring not only staff compliance, but importantly can be used to identify those who require additional support. By freeing up educational instructors, options are available for more effective use of their skills. Further, by reducing the reliance on instructors, education spaces and static simulation equipment, the simulation stations are able to better match the schedules of clinicians and reduce time away from direct patient care.

In this study, the BVM ventilation training was not superior under the automated manikin feedback condition in its current format. Further development of the manikin module with an improvement in task description, and differentiation between frequency and intensity of ventilation and monitoring, may be capable of reversing this finding [1, 4, 5, 9–24]. Psychomotor skills of ventilations are complex and require specific and timely feedback. One limitation for automated ventilation feedback concerns the underlying algorithm. Once the trainee has failed to meet the target ventilations, they are unable to recover within their session. That is, the underlying scoring system is not adaptive as the trainee makes adjustments to bag handling technique. In addition, the feedback timing is delayed so that adjustments that occur after the event may disrupt the attempt to self-correct. It is recommended that the automated feedback for ventilations is refined by Laerdal Medical, which may facilitate superior skills acquisition in this context.

Whilst the feedback condition did not impact participants’ confidence in the completion of the task, the majority reported that they found the automated manikin feedback more useful than the instructor feedback. Given this result, it would be interesting to determine if doctors would be more inclined to undertake regular training if they had access to an automated hospital life support training system.

## Caption Electronic Supplementary Material

1. Appendix A—Pre-training Survey

2. Appendix B—Post-training Survey

3. Appendix C—Documented automated manikin feedback

4. Appendix D—Hospital Life Support Assessment Form
