# Supplementary material for: A randomised trial of the effectiveness of instructor versus automated manikin feedback for training junior doctors in life support skills
Source: Perspect Med Educ. 2020 Nov 26;10(2):95–100. doi: 10.1007/s40037-020-00631-y (PMC7952489; doi:10.1007/s40037-020-00631-y)
Supplement: Supplementary file 1 — 1. Appendix A—Pre-training Survey [file 40037_2020_631_MOESM1_ESM.docx]

Thank you for participating in this brief survey.

The information that you provide in this survey will be used by the Medical Education Unit at Fiona Stanley Hospital (FSH) to improve hospital life support training.

All information that you provide in this survey is confidential and de-identified. It has no bearing on your employment status. The de-identified data may be published or presented for educational purposes.

# * 1. What is your age?


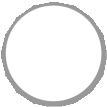
 Under 25 years


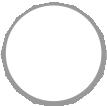
 25 to 29 years


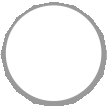
 30 to 34 years
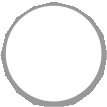
 35 years or older

# 2. What is your gender?


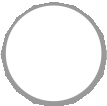
 Male
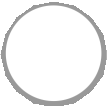
 Female

# 3. What postgraduate year (PGY) are you?


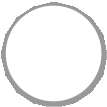
 PGY1
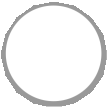
 PGY2
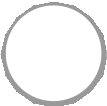
 PGY3
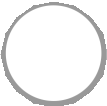
 PGY4+

# 4. Have you previously received hospital-based training in delivery of cardiac compressions and/or bag/valve mask ventilation?


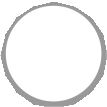

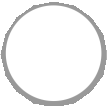
 Yes No

# 5. Did the hospital-based training in delivery of cardiac compressions and/or bag/valve mask ventilation involve the following feedback?


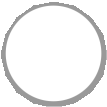

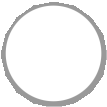
Yes No


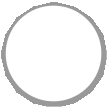

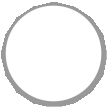


Instructor feedback

Voice advisory manikin feedback

# 6. Have you completed any acute care courses that included cardiac compressions and/or bag/valve mask ventilation skills, e.g. Resuscitation Council Advanced Life Support?


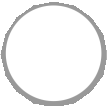

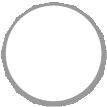
 Yes No

# 7. When did you last perform the following training?

Less than 3

months ago 4 to 6 months ago

7 to 12 months ago

More than 12

months ago Never


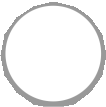

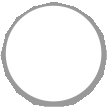

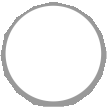

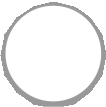

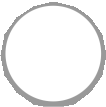


Cardiac compressions training


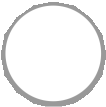

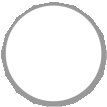

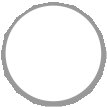

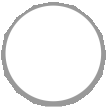

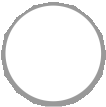
Bag/valve mask ventilation training

# 8. To what extent do you agree with the following statements?

Neither agree nor

Somewhat

Strongly agree Somewhat agree

disagree

disagree Strongly disagree


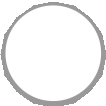

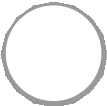

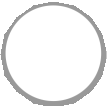

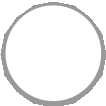

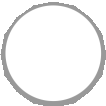


I am confident in my ability to deliver cardiac compressions in an acute setting

I am confident in my ability to deliver

ventilation using a
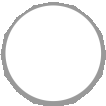

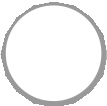

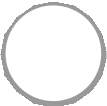

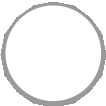

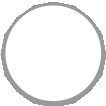


bag/valve resuscitation mask

Thank you for completing this survey.
