# Supplementary material for: A randomised trial of the effectiveness of instructor versus automated manikin feedback for training junior doctors in life support skills
Source: Perspect Med Educ. 2020 Nov 26;10(2):95–100. doi: 10.1007/s40037-020-00631-y (PMC7952489; doi:10.1007/s40037-020-00631-y)
Supplement: Supplementary file 2 — 2. Appendix B—Post-training Survey [file 40037_2020_631_MOESM2_ESM.docx]

Thank you for participating in this brief survey.

The information that you provide in this survey will be used by the Medical Education Unit at Fiona Stanley Hospital (FSH) to improve hospital life support training.

All information that you provide in this survey is confidential and de-identified. It has no bearing on your employment status. The de-identified data may be published or presented for educational purposes.

*** 1. To what extent do you agree with the following statements?**

Neither agree nor

Somewhat

Strongly agree Somewhat agree

disagree

disagree Strongly disagree


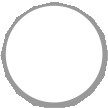

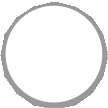

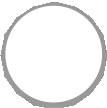

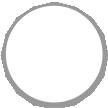

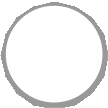


I found the voice advisory manikin feedback on the RQI cart more useful in comparison to the instructor feedback


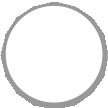

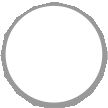

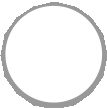

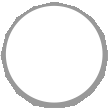

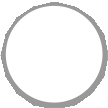
I am confident in my ability to deliver cardiac compressions in an acute setting


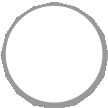

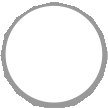

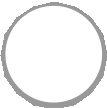

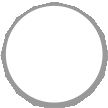

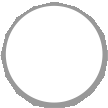


I am confident in my ability to deliver ventilation using a bag/valve resuscitation mask

Thank you for completing this survey.
