# Supplementary material for: A randomised trial of the effectiveness of instructor versus automated manikin feedback for training junior doctors in life support skills
Source: Perspect Med Educ. 2020 Nov 26;10(2):95–100. doi: 10.1007/s40037-020-00631-y (PMC7952489; doi:10.1007/s40037-020-00631-y)
Supplement: Supplementary file 3 — 3. Appendix C—Documented automated manikin feedback [file 40037_2020_631_MOESM3_ESM.docx]

**Appendix C. Documented automated manikin feedback phrases provided by the RQI**

| **Feedback phrases provided during cardiac compressions** | **Feedback phrases provided during BVM ventilation** |
| --- | --- |
| Place your hands a little higher on the chest | More air |
| Place your hands on the centre of the chest | A little more air |
| Compress a little deeper | Just a little less air |
| Use your full body weight | Ventilate a little less forcefully |
| Release pressure between compressions | Ventilate less often |
| Release | Ventilate more often |
| Compress a bit more often | Please let the manikin exhale completely before starting the next ventilation |
| Compress faster | Increase ventilation rate |
| Don’t compress so fast | Decrease ventilation rate |
| Great | Great |
| Good | Good |
| That’s right | That’s right |
| You are doing well | You are doing well |
