# Supplementary material for: A randomised trial of the effectiveness of instructor versus automated manikin feedback for training junior doctors in life support skills
Source: Perspect Med Educ. 2020 Nov 26;10(2):95–100. doi: 10.1007/s40037-020-00631-y (PMC7952489; doi:10.1007/s40037-020-00631-y)
Supplement: Supplementary file 4 — 4. Appendix D—Hospital Life Support Assessment Form [file 40037_2020_631_MOESM4_ESM.docx]

| Hospital Life Support Assessment Form | | |
| --- | --- | --- |
|  | |  |
| Group: Blue 1A |  | He Number: HE126490 |
|  | | |
| First Name: XXXX |  | Last Name: XXXX |
|  | | |
|  | | |
| Assessment Time: | |  |
|  | |  |
| Instructor feedback | | |
| Ventilation (75 seconds – Pass / Fail – provide reasoning) | | |
| Compressions (60 seconds – Pass / Fail – provide reasoning) | | |
| Instructor result: | | |
| RQI Result: | | |

| ASSESSMENT CRITERIA | |
| --- | --- |
| Delivery of high quality cardiac compressions | - Assess for 60 seconds - Maintains correct hand position   - Centre of chest, the lower half of the sternum - Compressions   - 5 cm depth (1/3 depth of chest)   - 100-120 compressions per min   - Avoid interruptions |
| Effective ventilation | - Assess for 75 seconds - Positioning   - Head tilt/chin lift manoeuvre   - Maintains good seal - Timing   - Aiming for 10 breaths per minute (range 8 – 12) - Volume   - 400mL – 500mL per breath - Flow Rate   - Does not rapidly deliver oxygen |
